# Supplemental 25-hydroxycholecalciferol Alleviates Inflammation and Cardiac Fibrosis in Hens

**DOI:** 10.3390/ijms21218379

**Published:** 2020-11-08

**Authors:** Pao-Chia Chou, Yu-Hui Chen, Thau-Kiong Chung, Rosemary L. Walzem, Lih-Shiuh Lai, Shuen-Ei Chen

**Affiliations:** 1 Department of Food Science and Biotechnology, National Chung Hsing University, Taichung 402, Taiwan; paochia88@yahoo.com.tw; 2 Department of Animal Science, National Chung Hsing University, Taichung 402, Taiwan; cute5409@yahoo.com.tw; 3 DSM Nutritional Products Asia Pacific, Singapore 117440, Singapore; Thau-Kiong.Chung@DSM.com; 4 Department of Poultry Science, Texas A&M University, College Station, TX 77843, USA; rwalzem@tamu.edu; 5 The iEGG and Animal Biotechnology Center, National Chung Hsing University, Taichung 402, Taiwan; 6 Research Center for Sustainable Energy and Nanotechnology, National Chung Hsing University, Taichung 402, Taiwan; 7 Innovation and Development Center of Sustainable Agriculture (IDCSA), National Chung Hsing University, Taichung 402, Taiwan

**Keywords:** 25-hydroxycholecalciferol, broiler breeder hens, inflammation, cardiac fibrosis, hypertrophy

## Abstract

Broiler breeder hens with efficient feed conversion rate under restricted feed intake (R-hens) or allowed unlimited access to feed (Ad-hens) progressed with cardiac functional failure and suffered early sudden death. A supplement of 69 μg 25-hydroxycholecalciferol (25-OH-D_3_)/kg feed improved heart health and rescued livability in both R- and Ad-hens throughout laying stage (26–60 wks). Improvements occurred through cardiac hypertrophic remodeling, reduced arrhythmias, and pathological cues. Here, we further demonstrated consistently decreased circulating and cardiac IL-6 and IL-1β levels in conjunction with reduced cardiac chemoattraction and leukocyte infiltration by 25-OH-D_3_ in Ad-hens and in R-hens at later time points (35 and 47 wks) (*p* < 0.05). Supplemental 25-OH-D_3_ also ameliorated cardiac fibrosis, endoplasmic reticulum (ER) stress, and autophagy, mostly in Ad-hens, as both collagen content and expression of COL3A1, as well as CCAAT box binding enhancer homologous protein (CHOP) and activating transcription factor 6 (ATF6), were consistently decreased, and suppression of microtubule-associated protein 1 light Chain 3 beta (LC3B) and Sequestosome 1 (SQSTM1) was rescued at 35 and 47 wks (*p* < 0.05). Vitamin D receptor-NF-κB signaling was shown to mediate these beneficial effects. The present results demonstrate that ER stress and autophagic processes along the sequence from inflammation to fibrotic changes contribute to pathological cardiac remodeling and functional compromise by Ad-feed intake. 25-OH-D_3_ is an effective anti-inflammatory and anti-fibrotic supplement to ameliorate cardiac pathogenesis in broiler breeder hens.

## 1. Introduction

Feed restriction (R) is commonly used to improve reproductive performance and livability in broiler breeder hens, due to the increased susceptibility to metabolic morbidities with genetic selection for rapid growth [1,2]. This approach, however, is under increasing scrutiny due to welfare concern of chronic hunger stress. Most studies of ascites and cardiomyopathy with respect to sudden death (SD) were carried out using growing juvenile broilers [3,4]. Few studies have examined the cause of mortality in adult broilers.

Previously, we showed a higher incidence of SD in broiler breeder hens provided with Ad libitum feed take (Ad-hens) and in R-hens with efficient feed conversion rates [1,2,5,6]. The mortalities were associated with peripheral hypertension; hyperglycemia; systemic inflammation; and dyslipidemia that provoked pathological cardiac remodeling, metabolic cardiomyopathy, arrhythmias, and finally functional failure [1,2]. To further elucidate the cause of SD and prevent SD, 69 μg 25-hydroxycholecalciferol (25-OH-D_3_)/kg feed was supplemented into the basal diet to examine its effects on cardiac health throughout the whole egg-laying stage. Results showed greatly improved livability in both Ad- (48.2% vs. 29.1%) and R-hens (86.7% vs. 78.9%) due to slowed progression of cardiac pathogenesis [5,6,7]. Among the SD-hens, 78.9%, 75.0%, 93.6%, 91.2% in the R, R+25-OH-D3, Ad and Ad+25-OH-D3 groups, respectively, were found with cardiac pathologies.

In a variety of animal models and clinical cases, vitamin D improved cardiac health and relieved functional failure by alleviating pathological hypertrophy, interstitial fibrosis, inflammatory status, and metabolic cardiomyopathy [8,9,10]. Vitamin D also limited cardiac pathogenesis by modifying systemic cues that include hypoxia, hypertension, and type-2 diabetes mellitus [11,12]. 

Cardiac fibrosis is a hallmark of various cardiovascular diseases such as myocardial infarction, and ischemic, dilated, and hypertrophic cardiomyopathies, which ultimately compromise cardiac contractile function [13]. Proinflammatory cytokines participate in cardiac hypertrophy, fibrosis, and cell apoptosis, contributing to ventricle dilation and chronic heart failure [14]. Based on those reports, this study aimed to examine the effects of 25-OH-D_3_ on cardiac inflammation and fibrosis in the development of hypertrophic remodeling in broiler breeder hens.

## 2. Results

### 2.1. Plasma IL-6, IL-1β Concentrations, and Cardiac Inflammation State and Chemotaxis

Release to allow Ad-feed intake caused a bust of feed consumption in 27 to 30 wks, approximately 35% and 10% more during 27 to 30 wks and 31 to 35 wks than the recommendation allocations by R-hens [5]. Supplementation of 25-OH-D_3_ had no significant effects on feed intake in both R- and Ad-hens [5].

**Figure 1:**
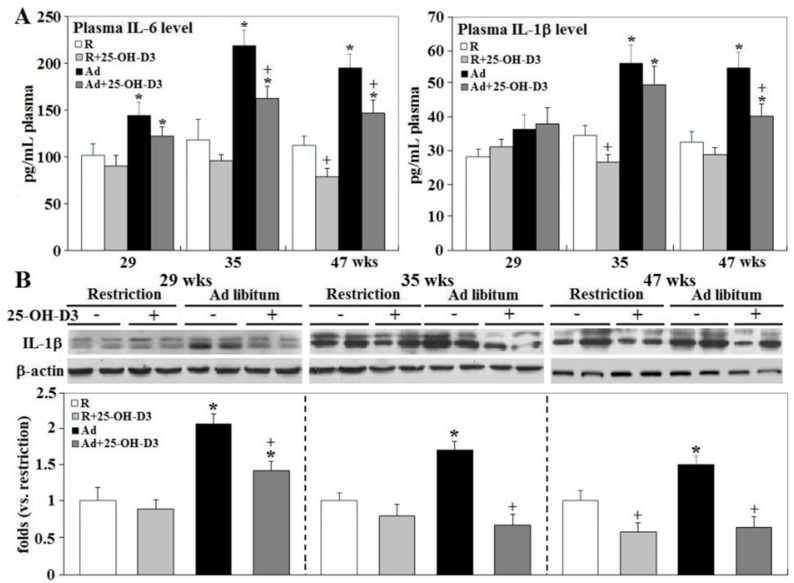
Effects of dietary 25-hydroxycholecalciferol (25-OH-D_3_) supplementation on systemic and cardiac inflammation of broiler breeder hens provided with restricted or ad libitum feed intake. Plasma IL-6 and IL-1β concentration were determined by ELISA method (*n* = 4, 7, 7 for each group at 29, 35, and 47 wks, respectively, panel (**A**). Four hearts from collections at 29, 35, and 47 wks were used for cardiac IL-1β analysis by Western blot (*n* = 4 for each group, panel (**B**). Densitometric intensities of Western blot were normalized to β-actin and expressed as ratios relative to R-hens at 29, 35, or 47 wks. *; significant difference by Ad-feed intake (*vs*. corresponding R hens, *p* < 0.05). +; significant difference by 25-OH-D_3_ (vs. R or Ad hens, *p* < 0.05). R; restriction, Ad; ad libitum, 25-OH-D_3_: 25-hydroxycholecalciferol.

**Figure 2:**
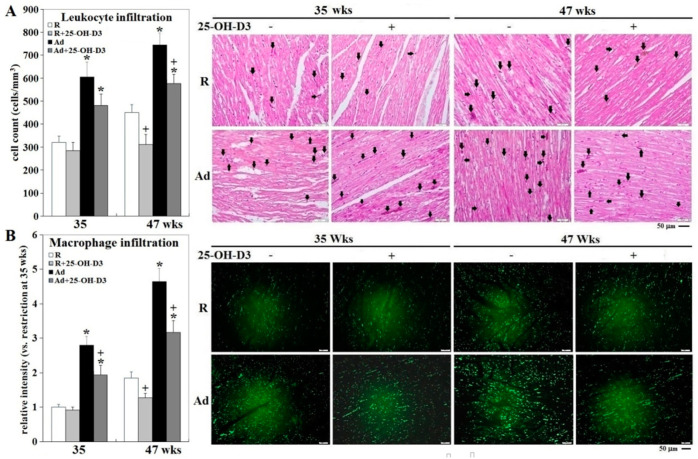
Effects of dietary 25-OH-D_3_ supplementation on cardiac leukocyte infiltration of broiler breeder hens provided with restricted or ad libitum feed intake. Three hearts from collections at 35 and 47 wks were used for histological and immunohistochemical studies (*n* = 3). Leukocyte infiltration was determined by counts in hematoxylin and eosin (H&E) staining (cells with intense purple color containing dark nuclei indicated by arrows, panel (**A**), and immunohistochemical analysis using a specific antibody against avian macrophages (panel (**B**)). Fluorescence results of macrophage infiltration were expressed as ratios relative to R-hens at 35 wks.*; significant difference by Ad-feed intake (vs. corresponding R hens, *p* < 0.05). +; significant difference by 25-OH-D_3_ inclusion (vs. R or Ad hens, *p* < 0.05). R; restriction, Ad; ad libitum, 25-OH-D_3_: 25-hydroxycholecalciferol.

**Figure 3:**
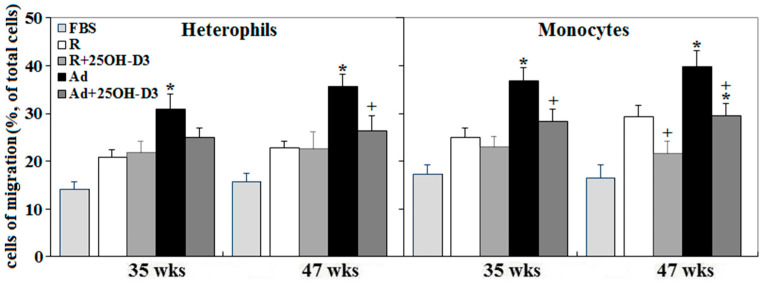
Effects of dietary 25-hydroxycholecalciferol supplementation on cardiac chemoattraction of broiler breeder hens provided with restricted or ad libitum feed intake. Four hearts from collections at 35 and 47 wks were used as a chemoattractant for leukocyte migration analysis through the trans-well method. Fetal bovine serum (FBS) served as a reference. Isolated leukocytes were pooled from 2 R-hens at age of 35 or 47 wks. *; significant difference by Ad-feed intake (vs. corresponding R hens, *p* < 0.05). +; significant difference by 25-OH-D3 inclusion (vs. R or Ad hens, *p* < 0.05). R; restriction, Ad; ad libitum.

In accordance with improved cardiac health and livability [5,6,7], both plasma IL-6 (interleukin 6) and IL-1β concentrations, and cardiac IL-1β expression, were decreased by 25-OH-D_3_ in Ad- and/or -R hens at 35 and 47 wks (*p* < 0.05, Figure 1). Concomitantly, Ad-feed intake dramatically promoted leukocyte infiltration, while supplemental 25-OH-D_3_ significantly reduced leukocyte infiltration in both Ad- and R-hens at 47 wks (*p* < 0.05, Figure 2). Cell migration analysis manifested cardiac inflammation state by Ad feed intake, as more heterophils and monocytes were attracted to infiltrate across the membranes by the chemoattraction of heart homogenates from Ad-hens (*p* < 0.05, Figure 3). Supplemental 25-OH-D_3_ suppressed cardiac chemoattraction in Ad-hens at 35 and 47 wks and R-hens at 47 wks (*p* < 0.05, Figure 3). 

### 2.2. Cardiac Fibrosis

**Figure 4:**
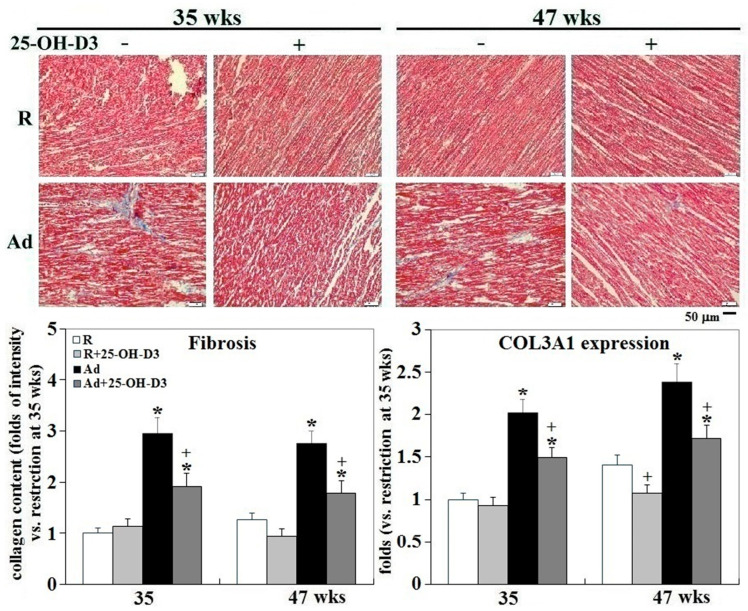
Effects of dietary 25-hydroxycholecalciferol supplementation on cardiac fibrosis of broiler breeder hens provided with restricted or ad libitum feed intake. Three hearts from collections at 35 and 47 wks were used for fibrosis analysis with collagen content by trichrome staining (blue color)(*n* = 3 for each group) and the remaining four hearts were used for COL3A1 (collagen type 3-𝛼1) expression analysis by qRT-PCR method (*n* = 4 for each group). Results of qRT-PCR and chromogenic intensity in trichrome staining were expressed ratios relative R-hens at 35 wks.*; significant difference by Ad-feed intake (vs. corresponding R hens, *p* < 0.05). +; significant difference by 25-OH-D_3_ (vs. R or Ad hens, *p* < 0.05). R; restriction, Ad; ad libitum, 25-OH-D_3_: 25-hydroxycholecalciferol.

Fibrosis is an essential component of tissue repair that serves to preserve tissue architecture; however, progressive fibrosis also reflects a pathologic state that results in scarring and impairments of architecture and contractile function [15,16,17]. Ad-feed intake persistently increased cardiac fibrosis and COL3A1 (collagen type 3-𝛼1) expression (*p* < 0.05, Figure 4). Supplemental 25-OH-D_3_ significantly reduced cardiac fibrosis and COL3A1 expression in Ad-hens at 35 and 47 wks, and in R-hens at 47 wks (*p* < 0.05, Figure 4).

### 2.3. Endomplasmic Reticulum (ER) Stress and Autophagy

**Figure 5:**
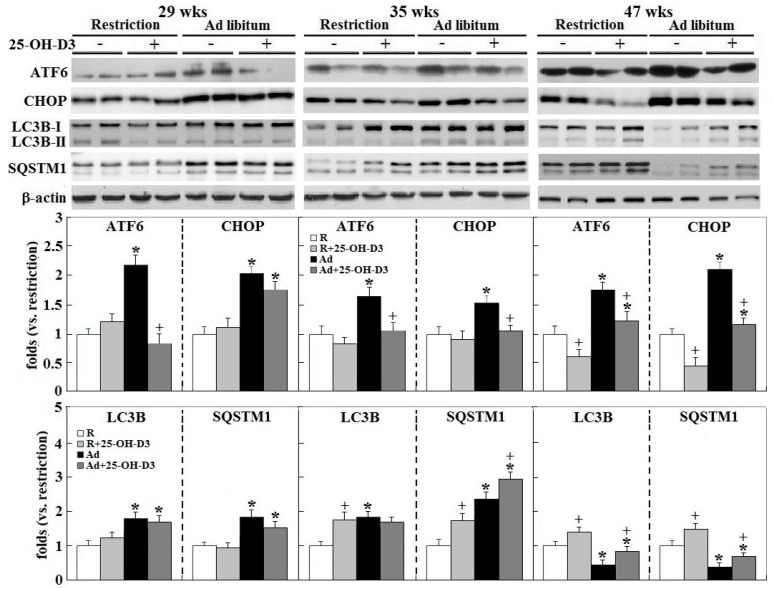
Effects of dietary 25-hydroxycholecalciferol supplementation on cardiac endoplasmic reticulum (ER) stress and autophagy of broiler breeder hens provided with restricted or ad libitum feed intake. ER stress and autophagy analysis includes activating transcription factor 6 (ATF6), CCAAT box binding enhancer homologous protein (CHOP), microtubule-associated protein 1 light Chain 3 beta (LC3B), and Sequestosome 1 (SQSTM1) by Western blot method (*n* = 4 for each group). Densitometric intensity results were normalized to β-actin and expressed as ratios relative R-hens at 29, 35, or 47 wks. *; significant difference by Ad-feed intake (vs. corresponding R hens, *p* < 0.05). +; significant difference by 25-OH-D_3_ (vs. R or Ad hens, *p* < 0.05).

Induction of autophagy exerts cardioprotective effects in several cardiovascular pathologies, and therefore reflects an adaptive mechanism by the heart in response to stress conditions. However, prolonged states of active autophagy may exacerbate the pathologies and cause detrimental results [18,19]. In the chicken model of obesity, progression of cardiac functional failure by Ad-feed intake [5,6] was associated with a consistent increase of cardiac activating transcription factor 6 (ATF6) and CCAAT box binding enhancer homologous protein (CHOP) expression and transiently increased microtubule-associated protein 1 light Chain 3 beta (LC3B), and Sequestosome 1 (SQSTM1) expression early in obesogenic feeding, but was significantly downregulated at the late stage in overtly obese hens (*p* < 0.05, Figure 5). In that setting, 25-OH-D_3_ supplementation significantly attenuated endoplasmic reticulum (ER) stress and rescued autophagic activity, as shown by ATF6 and CHOP expression along time course, and LC3B and SQSTM1 expression at 35 and/or 47 wks in Ad- and R-hens, respectively (*p* < 0.05, Figure 5).

### 2.4. VDR and NFκB Activation

**Figure 6:**
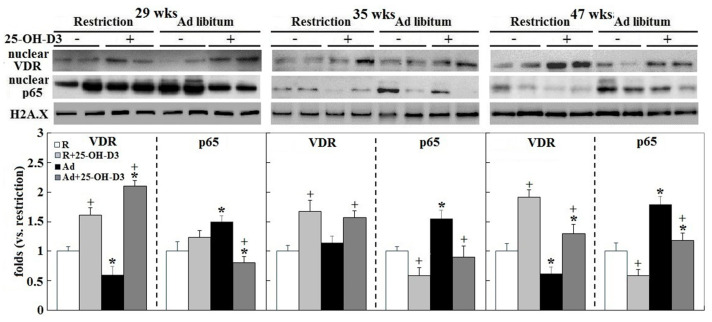
Effects of dietary 25-hydroxycholecalciferol supplementation on cardiac vitamin D receptor (VDR) and nuclear factor-κB (NFκB) activation of broiler breeder hens provided with restricted or ad libitum feed intake. Western blots of nuclear VDR and p65 (a subunit of NFκB; nuclear factor-κB) were assessed for (*n* = 4 for each group). Densitometric intensities results were normalized to histone family member X (H2A.X) and expressed as ratios relative R-hens at 29, 35, or 47 wks. *; significant difference by Ad-feed intake (vs. corresponding R hens, *p* < 0.05). +; significant difference by 25-OH-D_3_ (vs. R or Ad hens, *p* < 0.05).

Activation of vitamin D receptor (VDR) with nuclei translocation was decreased by Ad-feed intake at 29 and 47 wks, whereas nuclear factor-κB (NF κB) activation was increased along the time course (*p* < 0.05, Figure 6). Dietary inclusion of 25-OH-D_3_ consistently alleviated NFκB activation and promoted VDR translocation in both Ad- and R-hens (*p* < 0.05, Figure 6). The results confirmed VDR-NFκB signaling to mediate the beneficial effects by 25-OH-D_3_ on cardiac functions.

## 3. Discussion

In mammals, proinflammatory cytokines participate in the development of hypertensive end-organ damage such as the heart and kidney by provoking inflammation state as well as extracellular matrix remodeling [20,21,22,23]. Similar results, including systemic and pulmonary hypertension and cardiac pathological remodeling in association with increased inflammatory state and interstitial fibrosis, were also observed in broiler hens allowed Ad-feed intake [1,2]. We then demonstrated that dietary 25-OH-D_3_ supplementation ameliorated pathological cues, including peripheral hypertension and vascular remodeling, and relieved cardiac pathological remodeling and functional compromise [5,6,7]. Here, we further documented the beneficial effects of 25-OH-D_3_ on cardiac health by showing its ability to regulate inflammation state and fibrotic progression. These results as well as those from mammals suggest that vitamin D supplementation has the potential to limit the pathogenesis of diseases characterized by inflammation and fibrogenesis, such as cardiomyopathy and contractile failure [24].

Several endocrine and local stimuli such as hyperglycemia, elevated plasma levels of NEFA (non-esterified free acids), lipoproteins, oxidative stress, angiotensin-II, endothelin-1, and cytokine themselves have been shown to induce proinflammatory cytokine production by activating NF-κB pathway [25]. Activation of VDR by 1,25(OH)_2_D_3_ (1,25-dihydroxycholecalciferol) acts as a negative regulator in NF-κB signaling, due to VDR-p65 interaction and by interacting with IKKβ (IκB kinase beta) to maintain NF-κB sequestered by IκB (inhibitor kappa B) and thus prevent NF-κB translocation into the nuclei for downstream inflammatory gene expressions [26,27,28,29]. Moreover, in addition to NF-κB pathway, 1,25(OH)_2_D_3_ was shown to suppress high glucose-induced MCP-1 (monocyte chemoattractant protein-1) expression by directly upregulating VDR expression in both cell and animal models [30,31]. Accordingly, the present results confirmed the molecular aspect of VDR- NFκB signaling and upregulation of VDR expression to mediate 25-OH-D_3_ effects in the chicken model of obesity.

In a model of hypertension and end-organ damage by angiotensin II or high salt induction, deletion of IL-6 failed to prevent the development of hypertension, but attenuated myocardial inflammation and fibrosis, leading to an improvement in cardiac function [23]. Such observations suggest that proinflammatory cytokines accelerate the development of hypertensive heart diseases. However, a low constitutive level of IL-1β in the heart, induced by mechanical stretch, was sufficient to induce IGF-1 (insulin-like growth factor-1) production that negatively regulated JNK (c-Jun N-terminal kinase) signaling in the progression of cardiomyocyte hypertrophy from the compensated state to heart failure [32]. The counterbalance of hypertrophy-induced IL-1/IGF-1/JNK pathway thus determines the fate of the pressure-overloaded heart, namely, physiological compensative hypertrophy or maladaptive remodeling. Similar results, including sustained cardiac JNK activation and inflammation provocation in accompany with persistently elevated blood pressure, were also observed along the progression of pathological hypertrophy and functional compromise in the broiler breeder model [1,2]. Thus, improved inflammation status and fibrosis by 25-OH-D_3_ can be partially attributed to amelioration of mechanical loading in the heart due to its actions of the renin–angiotensin system and arterial remodeling that alleviate peripheral hypertension [7].

Cardiac hypertrophy reflects a compensatory response to enhance the function of the heart, yet active tissue remodeling processes triggered by various types of cues may diminish cardiac function. The major differences between the adaptive and maladaptive hypertrophy were associated with differential expression of genes involved in metabolism, fibrosis, and immune response [33]. Fibrosis is an essential component of tissue repair that follows tissue injury and is usually associated with inflammation [17]. However, progressive fibrosis due to chronic activation of fibroblasts by inflammation diminishes cardiac contractile function and causes structural damages [15,16,17]. 1,25(OH)_2_D_3_ was shown to inhibit cardiac myofibroblast activation and collagen synthesis by repressing TGF-β1 signaling [34]. Accordingly, 25-OH-D_3_ may act at the transition of cardiac remodeling by regulating inflammation state and fibrogenesis to decelerate the progression into maladaptive remodeling in Ad-hens. 

Parts of beneficial effects of vitamin D on cardiac pathologies, including hypertrophy, ischemia, arrhythmias, and heart failure, have been attributed to a relief of ER stress and related inflammation and cell apoptosis through VDR activation and downregulation of downstream targets, caspase, and CHOP gene expressions [35,36,37,38,39]. ATF6 and CHOP act as a mediator in ER stress-induced proinflammatory cytokine signaling [36,37,38]. Autophagy plays a dual role in the cardiovascular system depending on the strength of stress; physiological autophagy is protective and required to maintain normal cardiovascular function, while pathologic autophagy is involved in the manifestation of cardiovascular disease [36,40,41]. Consequently, sustained activation of autophagy by 25-OH-D_3_ during ER stress functions as an antiapoptotic process to prevent cell death by removing damaged proteins/organelles [41]. In diabetic rats, 1,25(OH)_2_D_3_ attenuated myocardial hypertrophy and interstitial fibrosis, improved cardiac function, and rescued cardiac autophagic activity through VDR activation and downstream β-catenin/TCF4/GSK-3β/mTOR (β-catenin/T-cell factor/glycogen synthase kinase-3β/mammalian target of rapamycin) pathway regulation [42]. Persistent hypertension combined with systemic and cardiac hypoxia and metabolic derangements by Ad-feed intake [1,2,5,6,7] thus ultimately caused failure of the heart’s cellular defense against mechanical and metabolic stresses that led to progressive pathological remodeling and finally functional compromise. Supplemental 25-OH-D_3_ protects the heart against pathological hypertrophy partially by operating at autophagic process and ER stress.

## 4. Materials and Methods

### 4.1. Animal Management

Cobb 500 broiler breeder hens at age of 22 wks purchased from a local breeder farm were fed to 26 weeks with a nutritionally adequate soy-and-corn-based breeder mash to achieve breeder company bodyweight recommendations [5]. All birds were caged individually within a house whose ambient temperature was maintained around 24–28 °C. Relative humidity varied with weather and was maintained between 55 and 85%. Birds had free access to water throughout the experiment. Feed was placed at 08:30 a.m. in conjunction with a 14L:10D (lights on at 05:00 a.m.) photo schedule. At age 26 weeks, 180 birds remained on restricted rations (R-hens) as recommended, while another 220 birds had sufficient feed for consumption to appetite (Ad-hens). Within each feed intake treatment, half of hens consumed a nutritionally adequate breeder diet, while the other half consumed this same diet containing additional 69 μg/kg feed of 25-OH-D_3_ (DSM Nutritional Products Ltd., Netherlands). Diets were fed until hens were 60 weeks old. The supplementation of 25-OH-D_3_ improved egg production due to its effects on livability [5,6]. Details of feed formulation, egg production, feed intake, and BW throughout the feeding trial were described previously [5]. The Institutional Animal Care and Use Committee of National Chung Hsing University, Taiwan approved all bird husbandry and procedures in accordance through an approved animal care protocol (IACUC Permit No. 102–113, 28 January 2017).

### 4.2. Necropsy and Tissue Collection

Hens were studied following an overnight fast and necropsied under anesthesia as described previously [43]. Organs and tissues (plasma, heart, liver, abdominal fat, lung, and pulmonary artery) were collected at ages 29, 35, and 47 wks, from 4, 7, and 7 randomly selected hens, respectively. Collected organs were used for gross pathological examination first. Four hearts from each group were used for molecular and biochemical studies, and the remaining 3 hearts collected at 35 and 47 wks were used for histochemical and immunostaining analysis. Collected liver and abdominal fat used for body compositional analysis and plasma, liver, lung, and pulmonary artery were also used for pathological studies, including morphological, molecular, and biochemical analyses [5,6,7].

### 4.3. Plasma IL-6 and IL-1β Concentrations and Cardiac Leukocyte Infiltration and Fibrosis

Plasma IL-6 and IL-1β levels were determined by validated commercial ELISA kits (Cat.# KMC0011, and KMC006; Biosource International, Camarillo, CA, USA, respectively) [44]. Paraffin embedded left ventricle sections were stained with (hematoxylin and eosin, H&E) to examine leukocyte infiltration or by trichrome Masson to visualize tissue fibrosis as described previously [1,2]. Macrophage infiltration was further visualized by antigen retrieval using an avian-specific mouse monoclonal antibody conjugated with FITC (Clone KUL01, Abcam, Cambridge, UK) [45]. Three sections per hen and 5 images from each section were used for intensity quantification using Image-J software (NIH, Bethesda, MD, USA).

### 4.4. Chemoattraction Analysis

Blood monocytes and heterophils were isolated using commercial Histopaque*^®^*1077 and 1119 (Sigma, St. Louis, MO, USA), and chemotactic migration of leukocytes was analyzed through the trans-well method as described in our previous studies [45,46]. In brief, 300 mg of ventricle were homogenized in 3 mL RPMI medium and then centrifuged at 15,000× *g* for 30 min at 4 °C. Pellets were collected and re-suspended in RPMI (10%, *w*/*w*). Peripheral heterophils or monocytes (1 × 10^5^ cells/well) pooled from 2 R-hens at age of 35 or 47 wks were plated onto the top trans-wells (pore size 5 μm). RPMI medium containing 10% (*w*/*w*) ventricle homogenates or 10% FBS (fetal bovine serum) as a reference was added to the lower chamber of trans-wells and allowed incubation for 4 h at 37 °C. After aspiration of medium, the lower chamber was fixed in methanol for 10 min, stained in Coomassie blue R250, and destained in 10% acetic acid. After drying, the membrane was mounted onto a slide and migrated cells were counted (five random fields per slide) under a microscope.

### 4.5. Western Blot Analysis

Left ventricle homogenates in RIPA buffer and nuclear extracts prepared using commercial kits (ab113474, Abcam) were used for Western blot analysis using a specific antibody against chicken IL-1β (Cat.# ab24771, Abcam) and antibodies cross-reactive to chicken antigens, including rabbit anti-SQSTM1/p62 (Cat.# ab101266, Abcam), anti-β-actin (Cat.# 4967), anti-p65 (Cat.# 8242, Cell Signaling Technology, Danvers, MA, USA), anti-CHOP (Cat.# sc-575), anti-ATF6 (Cat.# sc-166659, Santa Cruz Biotechnol. Inc., Dallas, TX, USA), anti-H2A.x (histone family member X, Cat.# PAB8764, Abnova Corporation, Taipei, Taiwan), anti-LC3B (Cat.# PM036, Medical & Biological Laboratories, Nagoya, Japan), and rat anti-VDR (Cat.# GR37-100UGCN, Merck, Kenilworth, NJ, USA). Horseradish peroxidase-conjugated secondary antibodies (Cell Signaling Technology) were used to identify the bands reactive to the primary antibodies.

### 4.6. Gene Expression by qRT-PCR

Freshly collected tissues were quickly dissected into 1 mm^3^ pieces on ice, dumped into RNA*later*^TM^ (Invitrogen, Waltham, MA, USA), and stored at –80 °C until use. Total RNA extraction, random priming reverse transcription, and qRT-PCR amplification were conducted as described previously [47] using commercial kits (Applied Biosystems, Waltham, MA, USA). Information about the primers is given in Supplement, Table S1. Reactions were conducted in triplicate, and the intra-assay CV (coefficient of variation) was less than 10%. 

### 4.7. Statistics

Data were analyzed by two-way ANOVA, in which feed intake (Ad or R) and 25-OH-D_3_ treatment were the classifying variables. Differences between group means were tested using Bonferroni corrected *t* test when the main effect was significant. If an interaction between feed intake and 25-OH-D_3_ treatment was found, a mean comparison was performed. Results were expressed as means ± SE. Mean differences were considered significant at *p* < 0.05. All statistical procedures were carried out using SPSS for Windows 13.0. 

## 5. Conclusions

Dietary supplementation of 25-OH-D_3_ relieved pathological cardiac remodeling and improved livability in broiler breeder hens with Ad-feed intake. Parts of the improvements were mediated by alleviation in cardiac ER stress and a sustained autophagic process in the sequence from inflammation to fibrotic changes.

## Supplementary Materials

The following are available online at https://www.mdpi.com/1422-0067/21/21/8379/s1, Table S1: Primers for quantitative real time polymerase chain reaction (qRT-PCR) analysis.
