# Supplementary material for: Supplemental 25-hydroxycholecalciferol Alleviates Inflammation and Cardiac Fibrosis in Hens
Source: Int J Mol Sci. 2020 Nov 8;21(21):8379. doi: 10.3390/ijms21218379 (PMC7664627; doi:10.3390/ijms21218379)
Supplement: Supplementary file 1 [file ijms-21-08379-s001.pdf]

Supplementary results

Table S1. Primers for quantitative real time polymerase chain reaction (qRT-PCR) analysis.

Gene	Access Number	Primer (5′-3′)	Amplicon (bp)	Optimized Primer Concentrations
**β-actin**	GI: 211236	F: CTGATGGTCAGGTCATCACCATT<br>R: TACCCAAGAAAGATGGCTGGAA	78	300 nM
**COL3A1 (collagen type 3-α1)**	GI: 966748959	F: GGTGGGCACACTTTCCTAAA<br>R: TTGAGCATTCAGGCAACAAG	102	500 nM

F: forward, R; reverse .
